# Flow cytometric characterisation of acute leukaemia in adolescent and adult Ethiopians

**DOI:** 10.4102/ajlm.v14i1.2394

**Published:** 2025-01-23

**Authors:** Jemal Alemu, Balako Gumi, Aster Tsegaye, Abdulaziz Sherif, Fisihatsion Tadesse, Amha Gebremedhin, Rawleigh Howe

**Affiliations:** 1 Department of Medical Laboratory Sciences, College of Health Sciences, Addis Ababa University, Addis Ababa, Ethiopia; 2 Armauer Hansen Research Institute, Addis Ababa, Ethiopia; 3 Aklilu Lemma Institute of Pathobiology, Addis Ababa University, Addis Ababa, Ethiopia; 4 Department of Internal Medicine, College of Health Sciences, Addis Ababa University, Addis Ababa, Ethiopia

**Keywords:** flow cytometry, acute leukaemia, Ethiopia, phenotype, cell surface biomarker, cytoplasmic biomarker

## Abstract

**Background:**

Flow cytometric characterisation of acute leukaemia is a key diagnostic approach for clinical management of patients, but is minimally practised in resource-constrained settings like Ethiopia.

**Objective:**

This study aimed to determine the immunophenotypes of acute leukaemia by flow cytometry at Tikur Anbessa Specialised Hospital, Addis Ababa, Ethiopia.

**Methods:**

A cross-sectional study was conducted on adolescent and adult inpatients consecutively admitted from April 2019 to June 2021. Peripheral blood samples were stained for surface and cytoplasmic markers, and analysed by four-colour flow cytometry.

**Results:**

Of 140 cases aged 13 years to 76 years, 74 (53%) were men and 66 (47%) were women, 68 (49%) had acute lymphocytic leukaemia (ALL), 65 (46 %) had acute myelogenous leukaemia (AML), and 7 (5.0%) had acute leukaemia non-otherwise specified. Acute lymphocytic leukaemia was more common among adolescent and male cases; AML was more common among adult and female cases. Among ALL subtypes, B-cell acute lymphocytic leukaemia cases (73.5%) were more common than T-cell acute lymphocytic leukaemia (26.5%). A subset of acute leukaemia, CD19+/CD56+ AML was identified in 3 cases (6% of AML). Of the B-cell ALL cases, 21 (42%) were CD34+/CD10+/CD66c+, 10% were CD34+/CD10+/CD66c–, 32% were CD34-/CD10+, and 6% were CD34+/CD10–. An unexpectedly high number of T-cell ALL cases that lacked surface CD3 were observed to have significantly higher levels of aberrantly expressed myeloid markers.

**Conclusion:**

We observed multiple phenotypes identifying subtypes of acute leukaemia cases, extending our previous studies in Ethiopia.

**What this study adds:**

This study extends previous studies by describing phenotypically defined subsets of ALL and AML which, in addition to diagnosis, may have useful prognostic value for clinicians.

## Introduction

Acute leukaemias are malignant disorders of precursor cells because of chromosomal rearrangements and multiple gene mutations, resulting in a high number of transformed cells within the bone marrow.^1,2^ Leukaemia is diagnosed according to clinical features, laboratory tests including morphological examination of blood smears, flow cytometry immunophenotyping, and cytogenetic and molecular analysis.^3,4^ Flow cytometry contributes important information to the assigning of leukaemic cell lineages, subtypes, and subsets in the diagnosis of and optimal therapy for acute leukaemia.^5^

Flow cytometric analysis determines expression patterns for cell surface and cytoplasmic molecules defining maturation stages, lineage, and subtype differentiation, as well as aberrantly expressed markers.^6^ While numerous studies have been conducted in developed countries on flow cytometric phenotyping of acute leukaemias, far less is known in resource-limited settings in East Africa, such as Ethiopia. Moreover, the method is not well established for routine care at the main tertiary care referral hospital of Ethiopia. Using this technology, we are continuing our efforts to integrate patient care with research and teaching. We previously conducted an initial research study^7^ showing the feasibility of this approach in this resource-limited setting.^8^ Our current work was aimed to determine the immunophenotypes of acute leukaemia by flow cytometry at Tikur Anbessa Specialised Hospital, Addis Ababa, Ethiopia.

## Methods

### Ethical considerations

Our study received approval from the Institutional Research Board of Aklilu Lemma Institute of Pathobiology, Addis Ababa University (reference no.: ALIPB IRB/009/2011/2018), the College of Health Science, Addis Ababa University (protocol no.: 001/19/IM), and the Armauer Hansen Research Institute (AHRI)/All Africa Leprosy, Tuberculosis and Rehabilitation Training Center (ALERT) Ethics Review Committee (protocol no.: PO34/18). Written consent was obtained from adult participants, whereas written assent was obtained from adolescent cases or their families/caregiver. Consent was also obtained from control participants. All personal information obtained from the study was coded to maintain complete confidentiality throughout the study period.

### Study design and setting

The present study was a cross-sectional study done at the haematology wards of Tikur Anbessa Specialised Hospital in Addis Ababa, Ethiopia, between April 2019 and June 2021. Tikur Anbessa Specialised Hospital is the largest referral hospital in the country for the diagnosis and treatment of malignancies.

### Study population and sampling strategy

The study includes 140 adult and adolescent (aged 13–17 years) inpatients, consecutively diagnosed with acute leukaemia and admitted to the adult haematology ward, who had not yet started chemotherapy and who were recruited for the study. Bone marrow samples from each patient had been obtained, and smears stained with haematoxylin and eosin were confirmed and classified as acute myelogenous leukaemia (AML) or acute lymphocytic leukaemia (ALL). The sample size of 140 was calculated by using the percentage of acute leukaemia cases (among total patients within a typical a 36-month period in the haematology ward) and the margin of error set at 5%, with level of confidence at 95% (Zα/2 = 1.96).

### Sample and data collection

Important socio-demographic as well as related clinical information was collected using a standardised form. Cases/families/caregivers were interviewed for demographic characteristics and clinical data were extracted from patient cards by haematology fellows as well as internal medicine residents under the supervision of the principal investigator and senior advisers.

In addition, we used test results from healthy individuals as positive controls for some of the markers as a quality control. Over 80 Tikur Anbessa Specialised Hospital laboratory staff members, AHRI research staff, or ALERT hospital laboratory staff volunteered a whole blood sample. We selected apparently healthy professionals by checking their complete blood count result with five differential count analysers and for the presence of abnormal results, including detection of immature cells. A total of three samples were rejected.

A peripheral blood sample of about 2 mL was collected from each patient by experienced and trained nurses, and transferred to tripotassium ethylenediaminetetraacetic acid vacutainer tubes (Becton Dickinson, Pont-da-Claix, France). The blood samples were sent to Tikur Anbessa Specialised Hospital main laboratory for complete blood count testing on an automated haematological analyser (Sysmex 2000i; Sysmex Corporation, Kobe, Japan) and retested for complete blood count (by Sysmex KH 21N; Sysmex Corporation, Kobe, Japan, or Coulter DXH 800; Beckman Coulter, Shirley, New York, United States) at the ALERT hospital, and the average values were taken.

Samples were transported on the same day to the AHRI immunology laboratory for flow cytometry analysis.

### Laboratory analyses

#### Flow cytometry

We used a previously published procedure for flow phenotyping.^7^ Briefly, for cell surface staining, 100 mL of blood was added to 5 mL polystyrene tubes to which various combinations of fluorescent-conjugated antibodies were added.^8^ The antibody target molecules and fluorochromes included: CD8-FITC, CD4-PE, CD45-PerCP-Cy5.5, CD3-APC, CD19-FITC, CD56-PE, CD33-FITC, CD34-PE, CD117-APC, CD13-FITC, CD10-PE, HLA-DR-APC, isotype control IgG1-FITC, isotype control IgG1-PE, isotype control IgG1-APC, CD66c-PE, CD15-FITC, CD14-FITC, CD7-PE, CD22-APC, and CD64-APC.

Mixtures containing CD8/CD4/CD3 and CD33/CD34/CD117 antibodies, and CD3, CD15, CD19, CD10, CD56 individual antibodies were obtained from Agilent Technologies (Glostrup, Denmark); the remaining monoclonal antibodies were received from Becton Dickinson (San Jose, California, United States). Antibodies were organised into 10 panels, typically using 4 antibody colours per panel. The same panel design was used for all leukaemia cases. This was done to assure uniformity among all subjects within the study as well as to ascertain potential aberrant expression of lineage-specific markers on some leukaemias, for example myeloid markers CD13, CD33, MPO, and CD66c on B-cell ALL (B-ALL).^8,9^

Cytoplasmic staining was performed with the following antibodies: CD45-PerCP-Cy5.5, terminal deoxynucleotidal transferase-FITC, cMyeloperoxidase (cMPO)-FITC, CD79a-PE, CD3-APC, IgG1-FITC, IgG1-PE, IgG1-APC. The cMPO/CD79a/CD3 mixture and terminal deoxynucleotidal transferase antibodies were obtained from Agilent Technologies (Glostrup, Denmark); the remaining antibodies were from Becton Dickinson (San Jose, California, United States). Staining was performed as described for phenotyping.^7^ Acquisition was performed with a FACS Calibur four-colour flow cytometer (Becton-Dickinson, Durham, New Hamshire, United States; an instrument that measures six parameters such as side scatter, forward scatter, and four types of fluorochrome-coated antibodies) using Cell Quest software (BD Biosciences, San Jose, California, United States), with typically 30 000 to 100 000 events acquired.

### Data analysis

Data were imported into an MS Excel (Microsoft, Redmond, Washington, United States) spreadsheet and thereafter exported to an MS Access (Microsoft, Redmond, Washington, United States) database where the data were reviewed, cleaned, and analysed with SPSS version 25 statistical software (IBM Corp, Armonk, New York, United States). A given marker was considered positive if > 20% of the leukaemia gated cells were above the cutoff threshold established by isotype controls separately for surface and cytoplasmic staining^11^; a representative gating example is depicted in Online Supplementary Figure 1.

In some instances, marker-positive cells were expressed as a percentage of gated leukaemia cells. In addition, for selected samples, marker density was calculated by dividing the mean fluorescence of marker-positive cells by that of marker-negative cells. The Chi-square test was used to determine associations between variables, and *p* < 0.05 indicates statistical significance; the Mann-Whitney U parametric test was used for continuous data.

#### Interpretation and grouping of acute leukaemia types and subtypes

Leukaemic cells were identified, typically (but not always) on the basis of reduced expression of CD45 and side scatter. Gated cells were then evaluated for expression of different markers. The threshold for marker positivity was determined by parallel samples stained with control isotype markers as described.^7^ Since markers typically were not entirely specific for a given lineage, we used World Health Organization (WHO)^10^ and Associazone Italiana Ematologia Oncologia Pediatrica and the Berlin-Frankfurt-Münster^11^ guidelines for AML, B-ALL, and T-cell ALL (T-ALL) classifications, and additional references for AML subtyping by flow cytometry.^12^

There are no generally accepted guidelines for the sub-classification of AML by flow cytometry aligned with the French-American-British categories, but we arbitrarily subdivided them into categories of AML ‘M0-2-like’ leukaemias (with phenotypes of either AML M0, M1 or M2), ‘M3-like’, ‘M4-like’ and ‘M5-like’, based on the percentage of cases with positive (> 20%) leukaemia cells and/or the percentage of leukaemia cells positive across those patients. Moreover, we provisionally defined cases that did not exhibit a straightforward lineage commitment, as acute leukaemia non-otherwise specified.^13^

## Results

### Demographic, clinical and flow cytometry characteristics

Out of a total of 140 patients, the ages ranged from 13 to 76 years, with a median of 25 (interquartile range: 18–35) (Table 1). Sixty-two per cent (46/74) of male patients and 54.5% (36/66) of female patients were within the age group of 18 years – 39 years (data not shown in table). No statistically significance associations were found between gender and acute leukaemia types or subtypes. The majority of patients clinically presented with anaemia (71.4%). Based on flow cytometry analysis, out of 140 cases, 68 (49%) were identified as ALL (B-cell and T-cell), and 65 (46%) as AML. Seven (5%) cases were acute leukaemia non-otherwise specified, although two cases appeared to meet requirements for mixed lineage (Table 1). Comparison of flow cytometry classification with independent classification into AML and ALL, using morphology of bone marrow smears, gave a concordance of 72%. Leukaemia and age were significantly associated (*p* < 0.001). Low haemoglobin and high white blood count levels were detected across all leukaemia types, without statistically significant differences. The highest platelet counts were found among T-ALL cases.

**TABLE 1:** Demographic, complete blood counts (haemoglobin, white blood count, and platelet count) and acute leukaemia types among adolescent and adult acute leukaemia patients before chemotherapy at Tikur Anbessa Specialised Hospital, Addis Ababa, Ethiopia, April 2019 to June 2021.

Variables	Acute leukaemia types																Total (*N* = 140)	*p*
	AML				B-ALL				T-ALL				AL-NOS					
	*n*	%	Median	IQR	*n*	%	Median	IQR	*n*	%	Median	IQR	*n*	%	Median	IQR		
**Gender**	-	-	-	-	-	-	-	-	-	-	-	-	-	-	-	-	-	0.077
Male	27	36.0	-	-	32	43.0	-	-	10	14.0	-	-	5	7.0	-	-	74	-
Female	38	58.0	-	-	18	27.0	-	-	8	12.0	-	-	2	3.0	-	-	66	-
**Age (years)**	65	46.0	-	-	50	36.0	-	-	18	13.0	-	-	7	5.0	-	-	140	< 0.001*
13 – 17	5	18.5	-	-	13	48.0	-	-	8	29.5	-	-	1	4.0	-	-	27	-
18 – 39	35	42.6	-	-	35	42.6	-	-	8	9.8	-	-	4	5.0	-	-	82	-
40 – 59	16	73.0	-	-	2	9.0	-	-	2	9.0	-	-	2	9.0	-	-	22	-
≥ 60	9	100.0	-	-	0	0.0	-	-	0	0.0	-	-	0	0.0	--		9	-
**Haemoglobin (gm/dL)**	-	-	7.9	7.0 – 8.8	-	-	8.3	6.9 – 9.6	-	-	8.8	7.8 – 10.2	-	-	7.7	7.0 – 8.3	-	0.45
**White blood count (× 10^3^ cells/µL)**	-	-	16.4	3.8 – 48.4	-	-	7.4	2.8 – 23.2	-	-	23.0	7.8 – 72.8	-	-	7.6	3.3 – 27.2	-	1.00
**Platelets (× 10^3^ cells/µL)**	-	-	31.0	10.5 – 55.5	-	-	21.0	10.0 – 48.5	-	-	40.0	26.5 – 129.0	-	-	20.0	17.0 – 91.0	-	0.01*

AML, acute myelogenous leukaemia; ALL, acute lymphocytic leukaemia; AL-NOS, acute leukaemia non-otherwise specified; B-ALL, B-cell acute lymphocytic leukaemia; T-ALL, T-cell acute lymphocytic leukaemia; IQR, interquartile range.

* Chi-square result, *p* < 0.05 indicates statistical significance.

### Expression of markers in acute leukaemia types, subtypes and subsets

Nearly all (96%) B-ALL patients were positive for all three B lineage markers (CD19, cCD79a, CD22). Importantly, none of the ‘B-cell specific’ markers was uniquely specific to B-ALL. A number of cases of T-ALL expressed myeloid markers. Acute myelogenous leukaemia was observed to be positive for CD117, CD34 and cMPO (Table 2). Among B-ALL, 42% were positive for the aberrantly expressed myeloid marker, CD66c, together with CD34 and CD10 (21/50), 10% with CD34+/CD10+/CD66c– (5/50), 6% CD34+/CD10– (3/50) and 32% CD34–/CD10+ (16/50). One of the CD34+/CD10–cases was CD66c positive, while of the CD34–/CD10+ subset, 29% were CD66c positive (Table 3). Among sCD3 positive T-ALL, the average percentage of sCD3 positive cells (79.4%) was less than the average cCD3 positive cells (99%), and sCD3 was an average of 2.8-fold lower in density than that of cCD3; this difference was not observed among mature T-cells in the same samples (data not shown). Notably, cMPO density among cMPO-positive T-ALL leukaemias was substantially 5.4 times less than that of cMPO-positive AML cases (*p* < 0.001) (Table 2).

**TABLE 2:** Percentage of patients who tested positive for indicated markers among acute leukaemia cases, Tikur Anbessa Specialised Hospital, Addis Ababa, Ethiopia, April 2019 to June 2021.

Marker	Acute leukaemia types (% of positive cases)			*p*
	B-ALL	T-ALL	AML	
CD4	16	50	43	< 0.001*
cCD3	4	100	5	< 0.001*
CD3	6	72	2	0.001*
CD19	98	6	17	< 0.001*
CD56	10	17	17	0.065
CD33	34	17	88	< 0.001*
CD34	68	39	77	< 0.001*
CD117	0	11	95	< 0.001*
CD13	50	17	85	< 0.001*
CD10	94	22	3	< 0.001*
HLA-DR	97	18	78	< 0.001*
CD15	18	28	28	0.014*
CD66c	60	14	22	< 0.001*
CD64	0	0	29	< 0.001*
CD14	2	0	8	< 0.001*
cCD79a	100	50	22	< 0.001*
CD8	16	50	3	< 0.001*
TdT	30	50	22	0.43
CD7	4	100	25	0.002*
CD22	98	11	15	< 0.001*
cMPO	20	30	91	< 0.001*

Note: Immunophenotypes of B-ALL (*n* = 50), T-ALL (*n* = 18) and AML (*n* = 65) leukaemia cases are depicted as percentage of patients positive for the indicated marker. Marker positivity for a given leukaemia means at least 20% of the leukaemia were positive for the given marker based on the gating strategy outlined in Online Supplementary Figure 1.

B-ALL, B-cell acute lymphocytic leukaemia; T-ALL, T-cell acute lymphocytic leukaemia; AML, acute myelogenous leukaemia.

* Chi-square result, *p* < 0.05 indicates statistical significance.

**TABLE 3:** Percentage of patients positive for indicated markers among B-cell acute lymphocytic leukaemia subsets at Tikur Anbessa Specialised Hospital, Addis Ababa, Ethiopia, April 2019 to June 2021.

Marker	B-ALL subset (Mean % of marker-positive leukaemia cells)			
	CD34– / CD10+ (*n* = 16)	CD34+ / CD10– (*n* = 3)	CD34+ / CD10+ / CD66c+ (*n* = 21)	CD34+ / CD10+ / CD66c– (*n* = 5)
CD19	67	74	76	74
CD33	21	51	15	13
CD34	7	91	65	86
CD13	19	52	35	23
CD10	82	10	89	89
HLA-DR	78	99	92	90
CD66c	20	14	63	24
CCD79a	87	81	86	85
TdT	41	1	47	15
CD22	72	70	82	87
MPO	2	5	28	3

Note: Immunophenotypes of B-ALL subsets. Forty-five of the fifty B-ALL cases were further stratified into subsets according to the expression of CD34, CD10 and CD66c (**17, 25).** Positive cases and negative cases were those with greater than or less than 20% marker-positive cells. Data shown represent the mean percentage of positive cells for a given B-ALL subset of patients for each marker (not the percentage of cases with > 20% positive cells).

B-ALL, B-cell acute lymphocytic leukaemia.

### Distribution of B-cell acute lymphocytic leukaemia subsets in age

When B-ALL subsets were stratified according to age, among B-ALL cases as characterised by CD34, CD10 and CD66 markers, 46% (6/13) of adolescents had a CD34+/CD10+/CD66+ phenotype and 8% (1/13) had a CD34+/CD10– phenotype (Table 4). Moreover, 41% (15/37) of adult cases had a CD34+/CD10+/CD66+ phenotype, while 5% (2/37) were CD34+/CD10–. Importantly, there were no statistically significant differences in the distribution of B-ALL subsets according to age (*p* = 0.93).

**TABLE 4:** Distribution of B-cell acute lymphocytic leukaemia subsets and acute myelogenous leukaemia subtypes among acute leukaemia patients by age group, Tikur Anbessa Specialized Hospital, Addis Ababa, Ethiopia, April 2019 to June 2021.

Acute leukaemia types	Age group of cases				Total	*p*
	Adolescents (13–17 years)		Adults (18 years and above)			
	*N*	%	*N*	%		
**B-ALL subsets**	-	-	-	-	-	0.930
CD34– / CD10+	4	31	12	32	16	-
CD34+ / CD10–	1	8	2	5	3	-
CD34+ / CD10+ / CD66+	6	46	15	41	21	-
CD34+ / CD10+ / CD66–	2	15	3	8	5	-
Others	0	0	5	14		-
**AML subtypes**	-	-	-	-	-	0.525
AML M0-M2-like	5	100	41	68	46	-
AML M3-like	0	0	10	17	10	-
AML M4-like	0	0	6	10	6	-
AML M5-like	0	0	3	5	3	-
Other	0	0	0	0	0	-

AML, acute myelogenous leukaemia; ALL, acute lymphocytic leukaemia; B-ALL, B-cell acute lymphocytic leukaemia.

When comparing sCD3 negative and T-ALL positive phenotypes, 20% of T-ALL cases without surface CD3 were positive for CD13, 30% were positive for CD33, and 42% were positive for MPO (Table 5). Among cases with sCD3 expressed, 8% were positive for CD13, 0% were positive for CD33, and 20% were positive for MPO.

**TABLE 5:** Differential expression of cell surface markers CD3 positive and negative T-cell acute lymphocytic leukaemia at Tikur Anbessa Specialised Hospital, Addis Ababa, Ethiopia, April 2019 to June 2021.

T-ALL subset	Average % positive cells		% of cases with > 20% marker-positive cells				
	sCD3	cCD3	CD10	CD56	CD13	CD33	MPO
sCD3 positive T-ALL (*n* = 13)	79.4	99	23	10	8	0	20
sCD3 negative T-ALL (*n* = 5)	6.3	87.4	20	60	20	30	42

T-ALL, T-cell acute lymphocytic leukaemia.

### Expression of markers in acute myelogenous leukaemia subtypes

**FIGURE 1:**
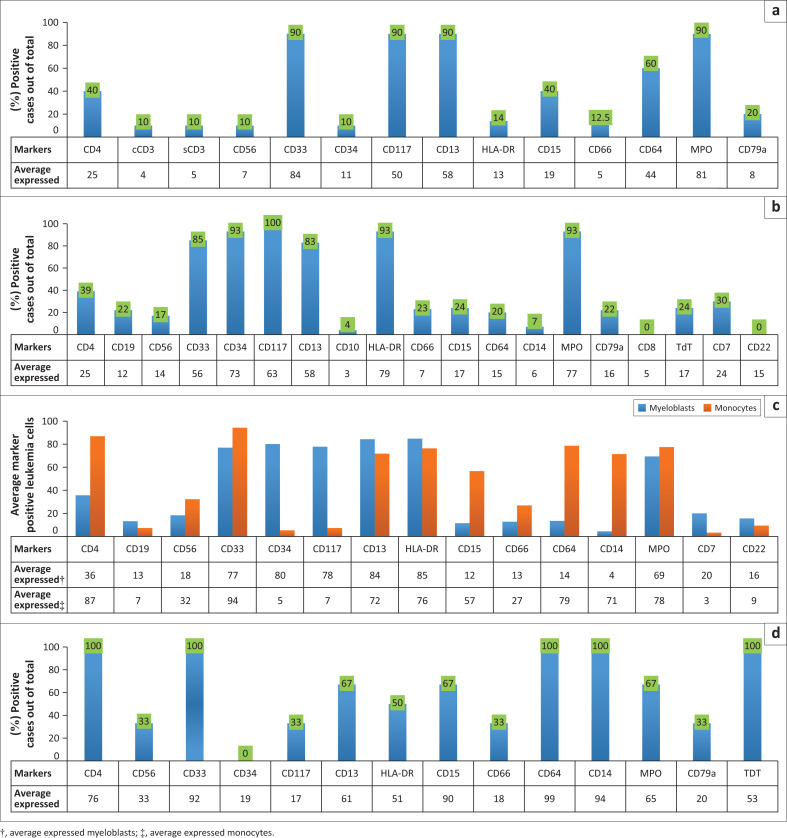
Immunophenotypes of arbitrary acute myelogenous leukaemia (AML) subtypes among acute leukaemia cases at Tikur Anbessa Specialised Hospital, Addis Ababa, Ethiopia, April 2019 to June 2021. (a) Immunophenotypes of AML-M3-like (*n* = 10), (b) AML-M0-M2-like (*n* = 46), (c) AML-M4-like (*n* = 6), and (d) AML-M5-like (*n* = 3) leukaemia cases. For (a), (b), and (d), markers are depicted either as percentage of cases with > 20% leukaemic cells (vertical bars) or percentage of leukaemia cells positive across all patients (indicated at the bottom row). For Figure (c), AML-M4-like, percentage of leukaemia cells positive across all patients is depicted for each of two separable populations, an immature myeloblast-like cell, and a more mature monocyte-like cell.

Among the 65 AML-like cases, 46 were thus arbitrarily classified as AML M0-2-like (71%), 10 as M3-like (15%), 6 as M4-like (9%) and 3 as M5-like (5%). Acute myelogenous leukaemia M0-2-like expressed markers of immaturity, 93% CD34, 100% CD117 and 93% HLA-DR positive, as well as other myeloid markers. Of note CD19 and CD56 were co-expressed in three cases (5%). Acute myelogenous leukaemia M5-like were typified by the demonstration of markers of mature monocytes CD64 and/or CD14, and lack of immature markers. Acute myelogenous leukaemia M4-like were notable for the presence of two distinct populations of leukaemia with differing densities of CD45 and side scatter, distinct from each other and from normal leukocytes, with one subpopulation bearing immature markers (CD34 and CD117), and the other monocyte markers CD14 and/or CD64. Acute myelogenous leukaemia M3-like cells were those intermediate between extremes of M0-2-like and M4-like or M5-like. These were typically CD117 positive and HLA-DR negative (Figure 1). Among AML subtypes, only the AML-M0-M2-like subtype was reported in adolescent cases (100% [5/5]), whereas all AML-like subtypes were reported in adult cases, including AML-M3-like, AML–M4-like and AML-M5-like (Table 4).

## Discussion

We observed a similar distribution of AML, B-ALL, T-ALL, as well as age associations, to our previous 2017 study^7^ and another African study.^14^ Secondly, we defined frequencies of subsets of B-ALL based on flow cytometric phenotypes having known associations with cytogenetic abnormalities, and find similarities consistent with previously reported prevalence. In particular, we observed a high prevalence of CD10+/CD34+/CD66+ B-ALL likely to be associated with t(9;22), and CD10+/CD34– B-ALL leukaemias, at least some of which have been associated with t(1:19) translocations.^15^ Thirdly, myeloid markers were preferentially expressed on T-ALL leukaemias with the most immature phenotypes. Fourthly, we report arbitrary flow cytometric classification of subtypes of AML based on a review of the study,^16^ and identify a subset expressing CD19 and CD56 consistent with the t(8;21) translocation. Collectively, these findings underscore the importance of multi-dimensional approaches for leukaemia diagnosis and prognosis.

We described subsets of B-ALL, using CD34, CD10 and, for some cases, CD66c, because these are commonly used markers and data exist describing their associations with clinical outcomes^17^ and with genetic aberrations.^18^ Moreover, CD34 and CD10 are well-described markers whose expression aids in delineating steps in early B-cell development.^19,20^ Thus, CD34+/CD10–, CD34+/CD10+ and CD34–/CD10+ represent successive stages of immature B-cell differentiation from pre-pro^19,20^ cells to, ultimately, transitional B-cells, although reports differ on the classification of intermediate pro-B and pre-B cells.^19,20^ Of note, CD34+/CD10+ B-ALL has also been termed Common B-ALL.^21^ These stages also appear to be similar to the B1, B2 and B3 subsets of B-ALL defined by the European Group for the Immunological Characterization of Leukemias classification,^22^ though reports differ on the classification of CD34+ positivity on pre-B cells.^23^ Including CD66c in the analysis allowed us to identify four subsets of B-ALL.

The CD34+/CD10+/CD66c+ subset was common in our study. A study in Saudi Arabia reported that the CD66c, CD34 and CD10 positivity phenotype were associated with, but not perfectly predictive of, the t(9;22) translocation, which is common in B-ALL, particularly among adults.^8^ Of note, CD66c positivity in B-ALL is also common in cases with hyperdiploidy.^24,25^ Among adults, it has a worse prognosis,^26^ but outcomes may be enhanced with the inclusion of tyrosine kinase inhibitors specific for the fusion gene *BCR-ABL*.^27^ Our finding that 40% of B-ALL in this cohort had the CD34+/CD10+/CD66c+ phenotype is consistent with another study.^25^

A second common phenotype in our study was CD10+/CD34–, consistent with another study.^9^ B-cell acute lymphocytic leukaemia with the t(1;19) translocation often has this phenotype, and reportedly with a favourable prognosis with intensive chemotherapy regimens.^28^ The prevalence of t(1;19) has been observed to be around 5% e,^28^ much lower than the associated phenotype that we report here. If the CD34–CD10+ phenotype is strongly predictive of t(1;19) aberrations, this implies that such aberrancies are exceptionally high in the Ethiopian setting. However, we favour the possibility that whereas most t(1;19) may be CD10+CD34–,^15^ the converse is not true, and there may be many other rearrangements or mutations present in this subset apart from the t(1;19) translocation. Further molecular work will be required to explore this possibility.

Less common in this study were two additional B-ALL phenotypes, the CD10+/CD34+/CD66c– phenotype, and the CD34+/CD10– subset (with or without CD66c). The CD10+/CD34+/CD66c– B-ALL subtype has been shown to be correlated with the t(12;21) translocation, which is the most common aberration in paediatric B-ALL, and which carries a good prognosis.^29^ In our cohort, the prevalence of this phenotype was low (10%), but our cohort was primarily composed of adult and adolescent leukaemia cases, so this lower frequency would be expected.^17^

The CD34+/CD10– subset has been shown to be associated with chromosome 11 mutations involving the *MLL* gene.^30^ The most common of these translocations is t(4;11) (MLL-AF4), present in 5% of ALL.^18^ Our finding of a 6% prevalence of the CD34+/CD10– phenotype is consistent with this.

We defined two populations of cytoplasmic CD3 expressing T-ALL, those without and those with detectable surface CD3 (sCD3). According to previous European Group for the Immunological Characterization of Leukemias classifications, the former would likely comprise the more immature T1 and T2, and the latter the less immature T3 and T4 phenotypes.^31^ Since we did not evaluate CD1 expression, we were unable to distinguish the cortical T3 from the more mature T4 phenotype. In our T-ALL cases, sCD3, when detectable, was variable in percentage, and typically of reduced intensity compared with peripheral normal T-cells. A notable difference between the sCD3 negative and positive subsets was the greater expression of myeloid markers CD33, cMPO and CD13, among the most immature T-ALL. cMyeloperoxidase expression is uncommon among T-ALL, although it has been described previously.^32^

We observed expression of myeloid antigens in B-ALL as well, confirming an other study.^33^ It is possible that some of the T-ALL and B-ALL described here may better fit the diagnosis of mixed phenotype acute leukaemia. This would imply a much higher prevalence of this subtype in the Ethiopian setting. However, weak expression of cMPO in particular is permitted within the classification criteria of Associazone Italiana Ematologia Oncologia Pediatrica-Berlin-Frankfurt-Münster^11^ for T-ALL, and we observed on average at least 4-fold lower levels of cMPO on those leukaemias we classified as T-ALL. From a mechanistic perspective, given that small fractions of early T-progenitor cells in normal individuals can express some myeloid markers,^16,34^ these leukaemias might persistently express incompletely repressed myeloid genes, as suggested for B-ALL.^33^ Further characterisation will be necessary to distinguish between these possibilities.

There are no generally accepted guidelines for the sub-classification of AML by flow cytometry. Originally, the French-American-British classifications defined subgroups of AML-M0, -M1, -M2, -M3, -M4, -M5, -M6 and -M7, which represented, respectively, AML with minimally differentiated, AML without maturation, AML with maturation, acute promyelocytic leukaemia, acute myelomonocytic leukaemia, acute monocytic leukaemia, acute erythroid leukaemia and acute megakaryocytic leukaemia. The newer WHO classifications^10^ comprise mostly recurrent cytogenetic translocations in AML, and AML not otherwise categorised. The former category includes AML with t(8;21), AML with t(16;16), AML with t(15;17), as well as several other translocations and genetic anomalies or mutations which are less common. The t(8;21) translocation is commonly associated with some AML-M2, the t(16;16) with some AML-M4, and the t(15;17) with some AML-M3. However, many genetic anomalies do not neatly map to old FAB categories, and, conversely, many AML do not have defined cytogenetic anomalies. The new WHO general category of AML not otherwise categorised thus includes subcategories which align with the previous FAB system.^10^ Because we were neither able to obtain cytogenetics for our samples, nor able to obtain FAB subtypes by morphology on most of the samples, we opted for an ad hoc flow cytometric classification based on published literature.^35^

There are extensive published data available, both in relation to FAB types as well as in relation to abnormalities.^35^ In the present study, we made arbitrary classifications based on these published data. With regard to AML-M0, -M1 and -M2, we made no attempt to differentiate between these types using flow cytometry. We further subdivided AML into M3-like, M4-like, M5-like categories. In comparing our distributions of AML subtypes with the reported literature our prevalence AML M0-2-like was higher than what would be predicted by other reports in the literature, and our estimated prevalence of M4-like and M5-like were lower.^36^ Among the four AML subtypes, all identified in adults and AML-M0-M2-like subtypes were more identified in adolescent cases, and it is similar with a previous study.^37^

In this study, M0-2-like was largely defined on the basis of the simultaneous expression of immature markers, including CD34, CD117 and HLA-DR. The majority of AML were of this arbitrary classification. However, CD34 and CD117 co-expression is not universal in AML-M0, -M1, and/or -M2.^35^ Thus, this may be a principal explanation why our prevalence estimates for AML subtypes differ from other studies.^38^

A distinct subset of AML-M2 leukaemias include those with the t(8;21) translocation, which typically results in the aberrant expression of CD19 and CD56, a phenotype which has been reported in association with the t(8;21) translocation.^39^ We observed three such cases in this cohort within our ‘M0-2-like’ subtype, numbers in line with predictions based on a study evaluating t(8;21).^39^ Notably, these three did not express other B-cell markers making a diagnosis of mixed phenotype or bi-lineage phenotype unlikely.^40^ A previous study described the t(8;21) translocations with lower density or lower percentage of positive cells expressing these two markers,^41^ and we did observe other ‘M0-2-like’ with similar phenotypes which could also conceivably express the t(8;21) translocation. Further work will be required to assess this possibility. In addition, we observed that among the ‘M0-2-like’ leukaemias, a relatively high fraction expressed the CD7 and TdT markers, typically associated with early lymphocyte progenitors and cells of the T-cell lineage, respectively. This was also observed in other subtypes of AML. The observation that such markers are commonly expressed among AML leukaemia is well documented.^42^

Acute myelogenous ‘M4-like’ leukaemia was easily visualised through the identification of immature myeloblasts and more mature monoblast-/monocyte-like populations.^12^ AML-M4 leukaemia is associated with the inv^16^ chromosome abnormality and also with bone marrow eosinophilia, leading to the unique WHO diagnostic classification. However, not all forms of AML-M4 express this abnormality,^43^ and other common abnormalities include CCAAT Enhancer Binding Protein Alpha (CEBPA) and NMP1 mutations,^44,45^ both with generally good prognosis.^46^

Acute myelogenous ‘M5-like’ leukaemia was primarily based in this study on the detection of monocyte-like cell subpopulations expressing CD14 and CD64 and lack of immaturity markers such as CD34 and CD117, though the latter two markers among M5 leukaemias have been cited in the literature.^40^ A number of mutations have been associated with M5, notably the MLL containing t(9;11)^47^ and also forms with NPM1 mutations,^44^ although both mutations are also seen in other AML types.^44^

The definition we used for AML ‘M3-like’ leukaemias represented those with phenotypes more intermediate to those of the immature marker expressing AML ‘M0-2-like’ and those expressing monocyte markers. Of note, the majority of ‘M3-like’ leukaemias in this study were CD34–/CD117+ and HLA-DR–, a flow cytometry phenotype often described in association with AML-M3.^48^ Importantly the AML-M3 phenotype is mostly associated with the t(15;17) rearrangement,^12^ but it is important to note that occasional t(15;17) translocations exist which are associated with other AML subtypes,^49^ and even markers such as cCD3.^50^

### Recommendations

Importantly, our study underscores the heterogeneity of leukaemia, and the need for future use of markers of molecular mutations or chromosomal arrangements, as well as evaluation of long-term patient outcomes. These are planned for future studies.

### Limitations

Our study was limited by the numbers of markers we utilised. In particular, we did not look for subsets of AML requiring additional markers to define erythroblastic or megakaryocyte leukaemia, mast, natural killer, basophil or dendritic cell leukaemias, and did not characterise mixed lineage or phenotype leukaemias. In addition, since morphology results from smears can only clearly distinguish between myeloid and lymphoid lineages, clear-cut comparisons cannot be made between morphology approaches and flow cytometry.

### Conclusion

Acute leukaemia diagnosis and prognosis represent a challenge in resource-limited settings. Most of the more advanced approaches utilised in developed country settings are unavailable; moreover, no single approach, even among advanced methodologies, is alone sufficient for diagnosis and prognosis according to current WHO guidelines. In the present study we have attempted to come up with a compromise approach, utilising more affordable four-colour flow cytometry and engaging in more detailed phenotypic characterisation of patient and leukaemia subtypes. However, it is clear that this approach, while promising, will require considerably more standardisation and validation in the context of WHO classification as well as patient outcomes.
